# Catalytic Performance of Ceria Nanorods in Liquid-Phase Oxidations of Hydrocarbons with *tert*-Butyl Hydroperoxide

**DOI:** 10.3390/molecules15020747

**Published:** 2010-02-02

**Authors:** Andreia G. Macedo, Sílvia E. M. Fernandes, Anabela A. Valente, Rute. A. S. Ferreira, Luís D. Carlos, João Rocha

**Affiliations:** 1 Department of Physics, CICECO, University of Aveiro, 3810-193 Aveiro, Portugal; E-Mail: agmacedo@ua.pt (A.G.M.); 2 Department of Chemistry, CICECO, University of Aveiro, 3810-193 Aveiro, Portugal; E-Mail: atav@ua.pt (A.A.V.)

**Keywords:** cerium oxide, nanorods, catalysis, oxidation, liquid phase, free radical mechanism

## Abstract

The CeO_2_ nanorods (CeNR) promote the oxidation of ethylbenzene (PhEt) and cyclohexene with t-BuOOH, at temperatures as low as 55 °C. For both substrates the saturated C-H bonds are preferentially activated over the unsaturated ones. The catalyst seems fairly stable towards leaching phenomena. The liquid-phase oxidation catalysis may be associated with the Ce**^3+^**/Ce**^4+^** inter-conversion in the one-electron redox processes mediating the formation of tert-butyl-(per)oxy radicals. CeNR is very effective in H**_2_**O**_2_** disproportionation. Pre-treatment of CeNR with H**_2_**O**_2_** or t-BuOOH prior to the catalytic reaction enhances the reaction rate of PhEt with t-BuOOH in comparison to CeNR. Textural characterization and spectroscopic studies suggest that catalytic activation is associated to defect sites.

## 1. Introduction

Cerium oxide has attracted much attention for several catalytic and environmental applications, such as depollution of noxious compounds from gaseous streams originating from industrial productions and from automobiles, the (industrial) gas-phase catalytic dehydrogenation of ethylbenzene to styrene, and the degradation of organic pollutants in wastewater through catalytic wet oxidation [1]. The interest arises from its remarkable Ce^3+^/Ce^4+^ redox and oxygen storage capacity properties. According to the literature the reduction of the ceria is controlled by the nature of oxygen vacancies, albeit the mechanism of creation of these favourable defects and their roles in the activation mechanisms, reducibility and activity of nanosized ceria at the atomic level is still lacking [2]. Cerium oxide has also been used in reactions for therapeutic applications to avoid reactive oxygen intermediates (ROI, like hydrogen peroxide) that increases retinal neurons degeneration (cerium nanoparticles were effectively used to scavenge these ROI within to retinal cells and inhibit the progressive degeneration) [3].The preparation route determines structural and surface properties of cerium oxide and there is a growing interest in controlling the morphology to obtain cerium oxide nanotubes. Very recently, cerium oxide nanotubes have been produced by hydrothermal alkali treatment [4], precipitation method [5], surfactant-assisted method [6], simple solid-liquid interface reaction route [7], by etching Ce(OH)_3_ nanotubes/nanorods with H_2_O_2_ [8] or using carbon nanotubes as templates by a liquid phase deposition method [9,10,11].

Studies of the influence of ceria materials in oxidation reactions are of interest since ceria is used as catalyst support in several oxidation applications. Here cerium oxide nanorods were tested as catalysts in the liquid phase oxidation of ethylbenzene using *tert*-butyl hydroperoxide (*t*-BuOOH) or H_2_O_2_ as oxidising agent: a possible reaction product is acetophenone which is used as ingredient in perfumes and as a chemical intermediate in the production of pharmaceuticals, resins and flavouring agents. Additionally, the catalytic oxidation of cyclohexene is investigated. The role of the Ce^3+^/Ce^4+^ redox and defect sites on the catalytic reaction is discussed. Additionally, the UV-vis absorption and luminescence techniques were used to study the presence and environment of Ce^3+^ in the samples before and after exposure to *t*-BuOOH or H_2_O_2_. The fluorescence of Ce^3+^ activated compounds is widely studied [12]. Usually, the Ce^3+^ emission consists of the allowed electric dipole *5d → 4f* transitions, since the Ce^3+^ has a 4*f* configuration; the ground states have a doublet with an energy separation around 2000 cm^-1^ (*^2^F_5/2_ and ^2^F_7/2_*). The lower excited states are the crystal field components of the 5*d* configuration. Also a broad band in a lower energy position has been attributed to the Ce4*f →*O2*p* charge transfer [13].

## 2. Results and Discussion

**Figure 1:**
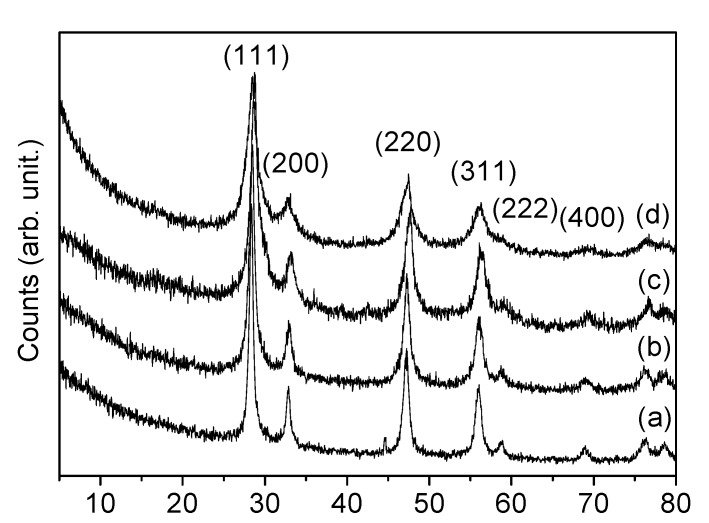
Powder XRD patterns of **(a)** CeNR, **(b)** CeNR-PhEt-BuOOH, **(c)** CeNR-BuOOH and **(d)** CeNR-H_2_O_2_.

**Figure 2:**
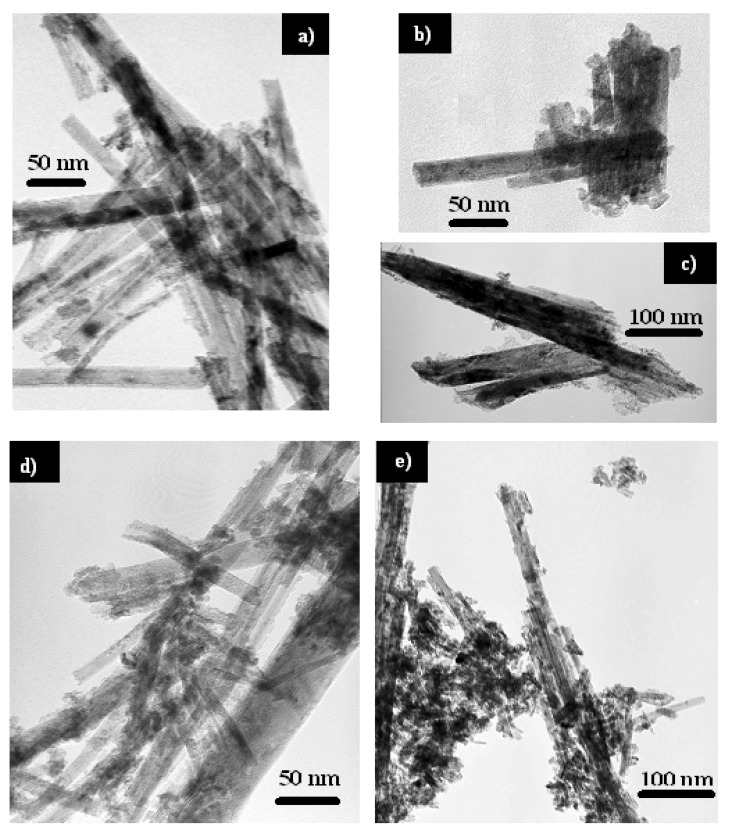
TEM images of **(a)** CeNR, **(b)** CeNR-BuOOH, **(c)** CeNR-H_2_O_2_, **(d)** CeNR-PhEt-BuOOH and **(e)** CeNR-PhEt-H_2_O_2_.

The powder XRD patterns of CeNR, CeNR-BuOOH, CeNR-H_2_O_2_ and CeNR recovered after catalysis (CeNR-PhEt-BuOOH-run2) are indexed to cubic fluorite-type CeO_2_ (JCPDS34-394) (Figure 1). The TEM shows that CeNR consists essentially of nanorods with a few tens of diameter and lengths of up to several hundreds of nanometers. Some fragmentation of the nanorods and formation of small 6–8 nm spherical nanoparticles occurred for CeNR-PhEt-BuOOH and CeNR-BuOOH and is much more significant for CeNR-H_2_O_2_ and CeNR-PhEt-H_2_O_2_ (Figure 2); this is consistent with ref. [5]. The formation of small nanoparticles is consistent with the broadening of the powder XRD reflections, which is clear in the case of CeNR-H_2_O_2_ (Figure 1).

**Figure 3:**
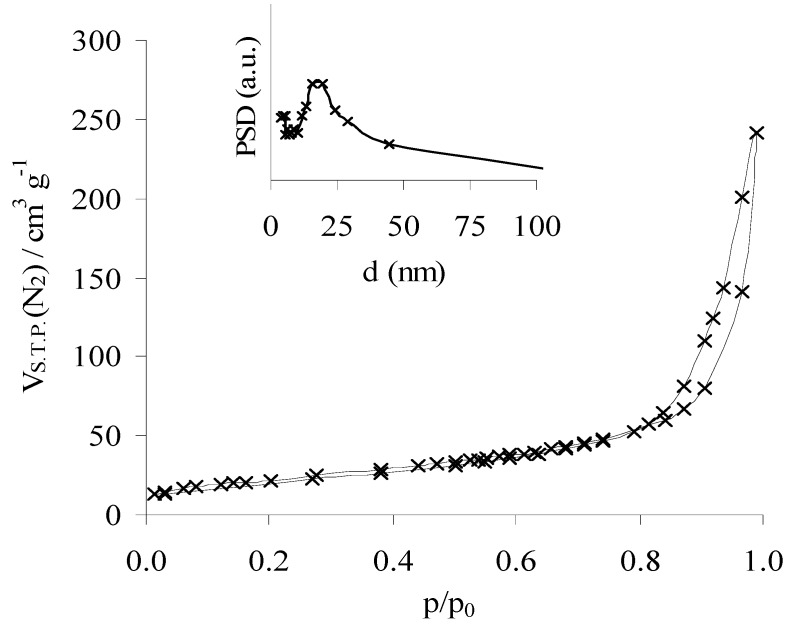
CeNR nitrogen adsorption-desorption isotherm (77 K) and (inset) pore-size distribution curve calculated for the desorption branch, using the Barrett-Joiner- Halenda method.

The nitrogen adsorption-desorption isotherm (-196 °C) of CeNR shows a substantial increase in the uptake of nitrogen at relative pressures greater than ca. 0.8, which is accompanied by hysteresis, possibly due to condensation/evaporation of nitrogen in/from the mesoporous network (Figure 3). The BET specific surface area is 78 m^2^/g (Table 1), and the PSD curve is rather broad with maximum pore widths of 16-20 nm (Figure 3): this may correspond to inter-particle voids and/or to open pores of a possible minor fraction of nanotubes. It is worth mentioning that the PSD curve calculated from the adsorption branch is not well-defined. The specific surface area of the pre-treated samples, CeNR-BuOOH and CeNR-H_2_O_2_ are comparable to that of CeNR.

### 2.1. Catalytic Oxidation of Ethylbenzene

#### 2.1.1. General

**Figure 4:**
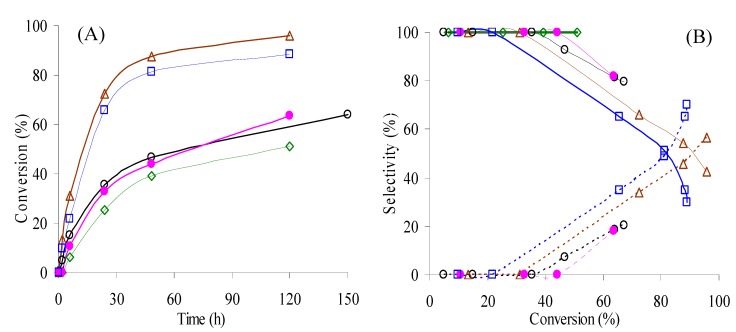
**(A)** - Ethylbenzene oxidation with *t*-BuOOH, in the presence of CeNR, at 55 °C (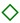), 70 °C (run 1 - (**O**); run 2 - (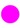)), 90 °C (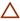), 105 °C (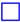), using acetonitrile as solvent. **(B)** - Dependence of selectivity to PhEtOOT (solid lines) and acetophenone (dashed lines) on conversion for the reactions conditions indicated in (A) - the same symbols were used for each reaction.

CeNR promotes the liquid-phase oxidation of ethylbenzene (PhEt), using *tert*-butyl hydroperoxide (*t*-BuOOH) as the oxidant and acetonitrile (CH_3_CN) as the solvent, at 55–105 °C. At 55 °C, 1-phenyl-ethyl-*tert*-butyl-peroxide (PhEtOOT) is the only reaction product until 51% conversion (reached after 120 h) (Figure 4): no reaction takes place without a catalyst. To the best of our knowledge, the formation of PhEtOOT in the catalytic oxidation of PhEt with *t*-BuOOH has not yet been reported. Syntheses procedures for PhEtOOT are based on the reaction of α-phenyl-ethyl bromide with potassium salt of *t*-butyl hydroperoxide [14]. In the range 70–105 °C, PhEtOOT selectivity decreases with PhEt conversion, while that to acetophenone increases (Figure 4B). These results suggest that acetophenone is formed from PhEtOOT. On the other hand, the PhEtOOT selectivity tends to decrease with the reaction temperature whereas that of acetophenone increases reaching 70% at 89% conversion, at 105 °C (Figure 4B, Table 2). Aromatic ring oxidation products were never detected revealing excellent regioselectivity of this catalytic system towards the side-aliphatic chain oxidation. The absence of carboxylic acids (which could be formed in consecutive oxidations) was checked by analysing derivatized samples by GC-MS.

**Table 1:** BET specific surface area.

Sample	S_BET_ (m^2^/g)
CeNR	78
CeNR-BuOOH	55
CeNR- H_2_O_2_	90

Increasing the temperature from 55 °C to 90 °C enhances the reaction rate: conversion at 6 h/120 h increases from 6%/51%, at 55 °C, to 31%/96%, at 90 °C (Figure 4A, Table 2). Increasing the temperature above 90 °C does not further increase the PhEt conversion. For the reaction carried out at 105 °C, the conversion of PhEt at 6 h/120 h is 22/88% and after 120 h remains nearly constant (89% at 175 h). Probably, the thermal decomposition (disproportionation) of *t*-BuOOH becomes a major competitive reaction for PhEt oxidation at high temperatures (see Section 2.1.3) [15].

When a radical scavenger (2,6-di-*tert*-butyl-4-methyl-phenol) is added initially to the reaction of PhEt with *t*-BuOOH in the presence of CeNT, at 70 °C, no reaction takes place, in contrast to that observed without the radical scavenger. These results indicate that a radical-chain process is involved. The initial induction periods of at least 120 min and 10 min observed for the reactions carried out at 55 °C and 70 °C, respectively (at higher temperatures no induction periods were detected), may be due to mechanistic factors. Possibly, the CeNR material acts as an initiator in the homolysis of *t*-BuOOH into free alkoxy (*t*-BuO^•^) and alkylperoxy radicals (*t*-BuO_2_^•^) *via* the net redox reaction represented in Equation 1: Ce^4+^ is a relatively strong one-electron oxidant, and the resulting Ce^3+^ species may be re-oxidized by *t*-BuOOH [16]. The *t*-BuO^•^ radical can abstract a proton from the substrate to give the corresponding radical (PhEt^•^), Equation 2 [17], which reacts with *t*-BuO_2_^•^ radicals to give PhEtOOT (Equation 3). It has been reported for the liquid-phase oxidation of arylbenzene compounds in homogeneous phase, in the presence of cerium ammonium nitrate/KBrO_3_ that radicals of the substrates may reduce Ce^4+^ into Ce^3+^ species, forming intermediate carbocations, which are subsequently oxidized [18]. Accordingly, cerium ions may promote the introduction of the alkylperoxy group of *t*-BuOOH into PhEt^•^*via* the net redox reaction represented in Equation 4. The reactions represented by Equations 5–6 are typically involved in radical chain processes of oxidation of hydrocarbons, using *t*-BuOOH as oxidant oxidant [16]. A control experiment showed that the oxidation of PhEt in the presence of CeNT and in the absence of *t*-BuOOH does not take place. Hence, the autoxidation of the substrate with molecular oxygen may be neglected (ethyl benzene hydroperoxide was not detected in the reaction mixtures by GC-MS): the observed induction periods may be due to intermediate rate limiting elementary steps.

The PhEtOOT product may be transformed into acetophenone (PhEt=O) plus *t*-BuOH *via* radical intermediates [14,17] (Equation 7). This hypothesis is supported by the fact that when CeNR is filtered from the reaction mixture after 24 h, at 70 °C (at this point PhEtOOT is the only product), and the obtained solution is left to react in the absence of CeNR until 48 h, at the same temperature, the formation of acetophenone is observed. On the other hand, the addition of a radical scavenger to the reaction of PhEt with *t*-BuOOH in the presence of CeNR, after 24 h, at 70 °C, not only inhibits the reaction of PhEt, but also no acetophenone is formed after 48 h, in contrast to that observed without a radical scavenger.


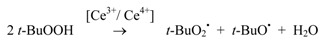
(1)

*t*-BuO^•^ + PhEt → *t*-BuOH + PhEt^•^ (2)

PhEt^•^ + *t*-BuO_2_^•^ → PhEtOOT (3)

PhEt^•^ + *t*-BuOOH → PhEtOOT + H^+ ^ (4)

*t*-BuO^•^ + *t*-BuOOH → *t*-BuO_2_^•^ + *t*-BuOH (5)

2 *t*-BuO_2_^•^ → 2 *t*-BuO^•^ + O_2_ (6)

PhEtOOT → PhEt=O + *t*-BuOH (7)

When 1,2-dichloroethane is used as solvent instead of acetonitrile, the reaction at 70 °C, gives 45% conversion at 48 h and PhEtOOT is the only product. These results are comparable to those observed for CeNR suggesting that a similar reaction mechanism is involved for both solvents. Further studies were carried out using acetonitrile a solvent.

#### 2.1.2. Stability of CeNR in the Liquid-Phase Oxidation Reaction

In order to get some insight into the stability of the CeNR under the applied oxidizing conditions, the solid was recovered from the reaction of PhEt with *t*-BuOOH, using acetonitrile as solvent, at 70 °C and used in a second batch run. The solid was separated from the reaction medium by centrifugation, washed consecutively with *n*-hexane, dichloromethane and acetone, and finally dried at room temperature prior to reuse (giving CeNR-PhEt-BuOOH). The kinetic curves for runs 1 and 2 are roughly coincident as well as the dependence of products selectivity on conversion (Figure 4, Table 2). The powder XRD patterns of CeNR and CeNR-PhEt-BuOOH are similar (Figure 1). The TGA analyses of the fresh and used solids gave similar weight losses between 30 °C and 600 °C (*ca.* 8 wt. %), under air, and therefore coking is negligible. These results suggest that CeNR is fairly stable under the applied oxidizing conditions.

Carrying out the reaction of PhEt in homogeneous phase in the presence of the precursor used for preparing CeNR, e.g., cerium sulphate (using the same mass as CeNR), at 70 °C, gives 15% conversion after 120 h, and PhEtOOT and acetophenone are formed in 34% and 66% selectivity, respectively (Table 2). The reaction is much faster in the presence of CeNT than that of Ce_2_(SO_4_)_3_.9H_2_O, under similar reaction conditions.

**Table 2:** Catalytic oxidation of PhEt with *t*-BuOOH, in the presence of the cerium-based catalysts.

Catalyst	Temperature (°C)	Conversion 6/120 h	PhEtOOT Selectivity 6/120 h	PhEt=O Selectivity 6/120 h
CeNR	55	6/51	100/100	0/0
CeNR (run 1)	70	15/64 ^a^	100/82 ^a^	0/18 ^a^
CeNR (run 2)	70	11/64	100/82	0/18
CeNR-BuOOH	70	16/97 ^a^	100/34 ^a^	0/64 ^a^
CeNR-H_2_O_2_	70	10/78	100/71	0/29
CeNR	90	31/96	100/43	0/57
CeNR	105	22/88	100/35	0/65
Ce(SO_4_)_2_.9H_2_O	70	0/15	-/66	-/34

^a^ Reaction time:150 h.

Commercially available CeO_2_ (powder, < 5 μm particle size, cubic fluorite-type, irregular morphology and apparently wide particle size distribution, Aldrich) gives 29% conversion after 150 h, at 70 °C, and PhEtOOT and acetophenone are formed in 63% and 37% selectivity, respectively; under similar conditions, the reaction is faster in the presence of CeNR (64% conversion), and PhEtOOT and acetophenone are formed in 18% and 82% selectivity, respectively.

In order to assess the homo/heterogeneous nature of the catalytic reaction in the presence of CeNR, the solid was separated by filtration (Whatman 0.2 μm PVDF w/GMF membrane) after 6 h reaction, at 70 °C, and the obtained solution was left to react at this temperature until 48 h. Conversion between 6 h and 48 h was approximately a third of that observed in the presence of CeNR (11% *vs.* 32%): possibly, the reaction proceeds in homogeneous phase due to (i) active CeNR, which was not separated by filtration and/or (ii) free radicals formed prior to catalyst filtration. Hypothesis (i) is ruled out by two experiments. The solid was separated from the reaction medium after 120 h by filtration at 70 °C, and the obtained solution was analysed by ICP-AES for cerium, which was not detected (detection limit *ca.* 5 ppb). Nevertheless, the obtained solution was evaporated to dryness and to the same vessel PhEt, *t*-BuOOH and CH_3_CN were added in the same amounts as those used for the first batch run: after stirring this mixture during 150 h, at 70 °C, no PhEt reaction was observed, indicating that the ‘residue’ obtained after evaporation did not contain active cerium compounds. Hypothesis (ii) is supported by the fact that hypothesis (i) is ruled out and the reaction stops when a radical scavenger is added, as mentioned in section 2.1.1. Based on these results, it seems clear that CeNR performs as a heterogeneous redox catalyst for generating free radicals, accounting for a radical chain process in the homogeneous phase.

When pristine CeNR was heated up to 600 °C in air no structural modifications were observed for the resulting solid (denoted CeNR600), as ascertained by powder XRD, SEM and FTIR (not shown). However, CeNR600 is inactive in the reaction of PhEt with *t*-BuOOH, at 70 °C. The calcination process induces marked differences in the colour (CeNR is yellow and CeNT600 is light maroon) and in the emission spectra of these materials, leading changes in the distribution of Ce^3+^/Ce^4+^ ions (as discussed ahead in section 2.3). The solid recovered (washed and dried) from the reaction of PhEt with *t*-BuOOH in the presence of CeNR, during 150 h, at 70 °C, was heated at 280 °C, during 3 h, under static air, giving a solid denoted CeNT280. The reaction of PhEt with *t*-BuOOH in the presence of CeNR280, at 70 °C, is slower than that observed for CeNR and CeNR-PhEt-BuOOH, and PhEtOOT is the only product until 54% conversion, reached after 120 h. Thermal treatments seem to gradually destroy the active sites, which may be defect sites in the original material. It has been reported in the literature that defect sites of the type Ce^3+^-O-Ce^4+^ are responsible for activating PhEt using N_2_O as oxidant, at 325 °C, and increasing temperature of H_2_ treatment from 300 °C to 350 °C led to a drastic decrease in concentration of these defect sites [19]. UV-vis and photoluminescence studies revealed the presence of both Ce^3+^ and Ce^4+^ in CeNR before and after it exposure to *t*-BuOOH, as discussed ahead.

#### 2.1.3. Oxidant Effect

When H_2_O_2_ (30% aq.) is used as oxidant instead of *t*-BuOOH, no PhEt reaction is observed in the presence of CeNR during 150 h, at 70 °C. It was previously reported for a ceria possessing a cubic crystal structure of the fluorite type (67 m^2^ g^-1^, 12 nm crystallite size), used as catalyst in the oxidation of PhEt with H_2_O_2_ and acetonitrile as solvent, that the reaction is sluggish giving *ca.* 4% conversion after 6 h, at 60 °C, and benzaldehyde and acetophenone were the only products [20]. The decomposition of H_2_O_2_ has been used as a test reaction for investigating the redox properties of ceria materials [1]. In the case of the CeNR/H_2_O_2_ system, the poor catalytic results may be due to the relatively fast ‘non-productive’ decomposition of H_2_O_2_ by CeNR. In order to investigate this hypothesis, iodometric titrations were performed for the reactions of CeNR with H_2_O_2_ or *t*-BuOOH (without PhEt) after 4 h, at 70 °C. These results revealed that H_2_O_2_ was totally consumed, whereas *t*-BuOOH conversion after 4 h was negligible, supporting the above hypothesis. It is worth mentioning that acetamide, which may be formed from the reaction of CH_3_CN with H_2_O_2_*via* the intermediate formation of the correspondent peroxyimidic acid was not detected in the reaction mixtures [21]. According to the literature, CeNR may undergo morphological changes in the presence of H_2_O_2_ [8]. A similar destructive effect of H_2_O_2_ has also been reported for cerium-containing SBA-15, in that cerium was removed from the silica framework, forming fine CeO_2_ crystallites on the surface [22].

To the best of our knowledge, the influence of *t*-BuOOH on the physico-chemical properties of CeNR has not been reported. In order to get some insight into the effect of the oxidant on the catalytic activity, the solids used in the reactions of PhEt with *t*-BuOOH or H_2_O_2_, at 70 °C, were recovered after 120 h in a similar fashion to that described above for the catalyst recycling experiments, and subsequently characterized: the resulting solids are denoted CeNR-PhEt-BuOOH and CeNR-PhEt-H_2_O_2_, respectively. On the other hand, for comparative studies, CeNR was treated with H_2_O_2_ or *t*-BuOOH, in the absence of PhEt, in an ultrasonic bath, as described in the experimental part, giving CeNR-H_2_O_2_ and CeNR-BuOOH, respectively. The TEM images of CeNR-BuOOH and CeNR-PhEt-BuOOH show some fragmentation of the nanorods and the formation of 6–8 nm spherical particles, and this effect is major for CeNR-H_2_O_2_ and CeNR-PhEt-H_2_O_2_ (Figure 2). The ATR-FTIR spectra of CeNR-BuOOH and CeNR-H_2_O_2_ are similar to that of CeNR in the region 1300–1600 cm^-1^, with the main difference being that the band at ca. 1640 cm^-1^ assigned to H-O-H bending vibration is very weak in the case of CeNR-BuOOH (not shown).The powder XRD patterns of CeNR-H_2_O_2_ and CeNR-BuOOH are similar to that of CeNR (see introduction of the section 2), albeit the peaks are slightly broader, especially in the case of CeNR-H_2_O_2_ (Figure 1).

**Figure 5:**
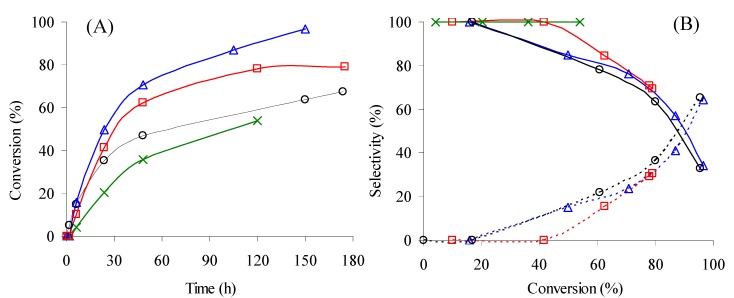
**(A)** - Ethylbenzene oxidation with t-BuOOH, at 70 °C, using acetonitrile as solvent, in the presence of CeNR (**O**), CeNR calcined at 280 °C (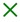),CeNR pre-treated with H_2_O_2_ (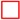) or *t*-BuOOH (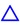). **(B)** - Dependence of selectivity to PhEtOOT (solid lines) and acetophenone (dashed lines) on conversion for the reactions conditions indicated in (A) - the same symbols were used for each reaction.

In order to assess the effect of the oxidizing pre-treatment on the reaction of PhEt, the CeNR-H_2_O_2_ and CeNR-BuOOH materials were tested as catalysts in the oxidation of PhEt with *t*-BuOOH, at 70 °C. No major differences in products selectivity were observed between the pre-treated solids and CeNR, at least until *ca.* 70% conversion, suggesting that a similar reaction mechanism is involved (Figure 5). However, the reaction rate (based on conversion at 24 h/120 h) is higher for the pre-treated materials than for CeNR, increasing in the order CeNR (35%/~60%) < CeNR-H_2_O_2_ (42%/78%) < CeNR-BuOOH (50%/~90%), Table 2. Altogether, the results support the hypothesis that CeNR is not effective in the reaction of PhEt with H_2_O_2_ because of its effectiveness in H_2_O_2_ disproportionation. The reaction of *t*-BuOOH on the surface of CeNR may primarily involve the coordination of the oxidant to defect sites (possibly co-ordinately unsaturated cerium), followed by redox reactions to form radical species, according to the above mechanistic considerations.

### 2.2. Oxidation of Different Substrates

CeNR was further tested as catalyst in the oxidation of cyclohexene and cyclohexanol, at 55 °C, during 24 h (Table 3). For both substrates, when H_2_O_2_ was used as oxidant, conversions were negligible, similar to that observed for PhEt. These results contrast with those reported for mesoporous Ce-SBA-15 in the oxidation of cyclohexene and cyclohexanol with H_2_O_2_, under similar conditions to those used in the present work: 40% cyclohexene conversion was reached after 10 h, at 50 °C, forming the epoxide as the main product (other products included the 1,2-diol, -en-1-ol and -en-1-one) and, on the other hand, the reaction of cyclohexanol with H_2_O_2_ gave a maximum of ca. 15% conversion after 7 h, at 60 °C [22]. The competitive ‘non-productive’ decomposition of H_2_O_2_ is probably less important for the referred supported ceria catalyst than for CeNR. Differences in catalyst surface polarity may lead to different competitive adsorption effects, which may partly explain the different catalytic performances of CeNR and Ce-SBA-15 using H_2_O_2_ as oxidant.

The reaction of cyclohexene with t-BuOOH in the presence of CeNR gives 22% conversion after 24 h and the main product is 1-(*tert*-butyl-peroxy)-2-cyclohexene (86% selectivity): by-products include 2-cyclohexen-1-one and minor amounts of 2-cyclohexen-1-ol (when no catalyst is added, no reaction is observed). The products selectivity for cyclohexene somewhat parallels that obtained for PhEt, in that for both substrates the major product is a *tert*-butyl-peroxy (alkyl or aryl) compound, and saturated C-H bonds are preferentially activated over the unsaturated ones. The 1-(*tert*-butyl-peroxy)-2-cyclohexene product has been identified in the oxidation of cyclohexene with *t*-BuOOH *via* radical reactions in the presence of free and alumina-supported transition metal complexes bearing Schiff base ligands [23,24,25].

The reaction of cyclohexanol with *t*-BuOOH gives cyclohexanone as the only product in 33% yield, after 24 h, at 55 °C (when no catalyst is added, cyclohexanone yield is 11%), Table 3. Based on these results the choice of a solvent for the catalytic oxidation reactions should preferably not include alcohols. For all substrates, CeNR600 gave negligible conversions, similar to that observed for PhEt.

**Table 3:** Oxidation of different substrates with t-BuOOH, in the presence of CeNR, using acetonitrile as solvent, during 24 h, at 55 °C.

Substrate	Conversion (%)	Product	Selectivity (%)
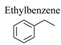	25	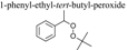	100
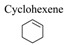	22	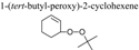	86^a^
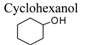	33	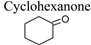	100

^a^ By-products are mainly 2-cyclohexen-1-one and minor amounts of 2-cyclohexen-1-ol.

### 2.3. UV-Vis and Photoluminescence Spectroscopy

**Figure 6:**
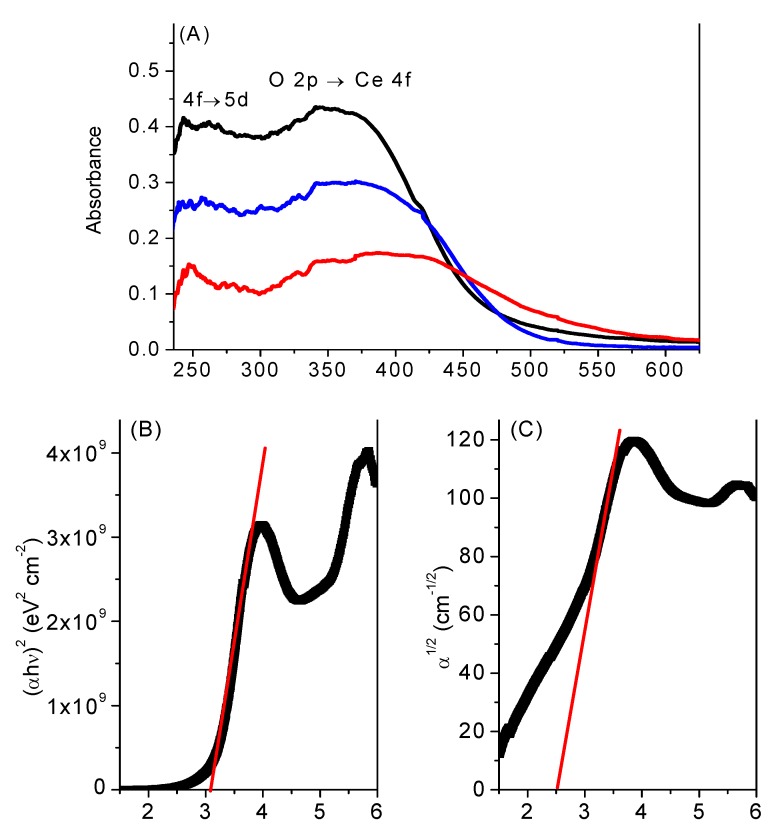
UV-Vis absorbance spectra of **(A)** CeNR (black), CeNR-BuOOH (blue) and CeNR-H_2_O_2_ (red) powders. Plots of **(B)** (αhν)^2^ versus (hν) and **(C)** α^1/2^ versus (hν) for the CeNR ethanol suspension.

**Figure 7:**
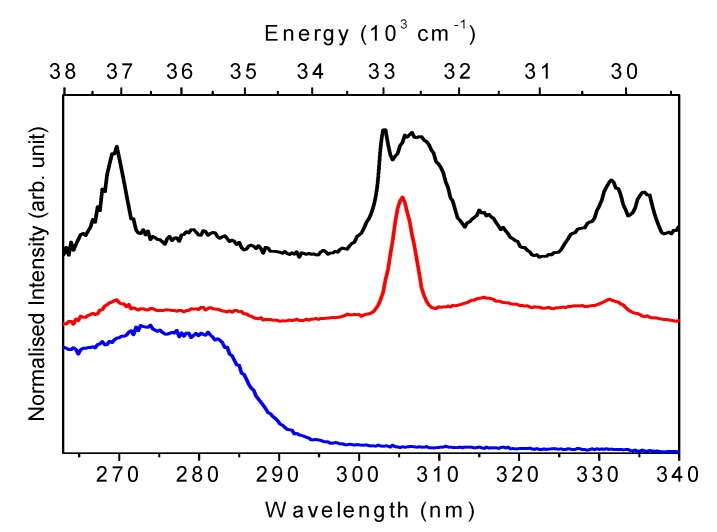
−11 K excitation spectra of CeNT monitoring at 370.1 nm (black), 390.2 nm (red) and 450 nm (blue).

Figure 6A displays the UV/Vis absorbance spectra of the as-prepared CeNR, CeNR-BuOOH, and CeNR-H_2_O_2_ powders. The peaks attributed to the 4*f*→5*d* transitions of Ce^3+^ (220–320 nm), are observed in all spectra. The excitation spectra (Figure 7) also exhibit Ce^3+^ 4*f*→5*d* transitions. A broad band (cut-off wavelength 480–550 nm) is also observed due to charge transfer transitions from O 2*p* to Ce 4*f* [27,28,29,30]: a broadening of this band is observed especially in the case of CeNR-H_2_O_2_ relative to CeNR possibly due to the increasing number of surface defects [13]. The optical energy gap, ε_d_, and the indirect band gap, ε_i_, were calculated for a suspension of CeNR in ethanol (Figure 6B and 6C). The values obtained (ε_d_ = 3.1 eV and ε_i_ = 2.5 eV) are in accord with the band gaps of ceria bulk [31,32]. The band gap values were not estimated for Ce-BuOOH and CeNR-H_2_O_2_ due their low dispersion and stability in suspension. However, the similarity of the absorption spectra measured for the CeNR and CeNR-BuOOH powders (Figure 6A) suggest that the energy gaps of both materials may be comparable, albeit may differ somewhat from that of CeNR-H_2_O_2_.

**Figure 8:**
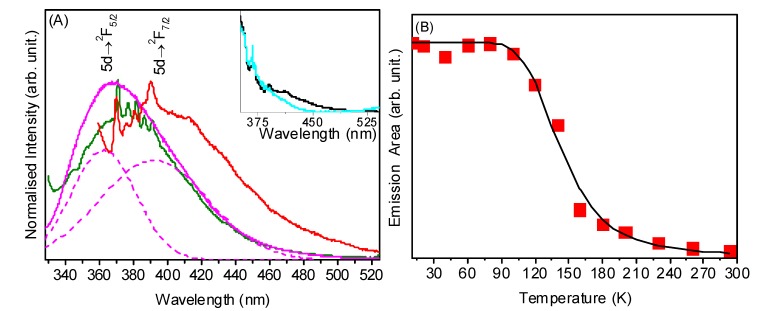
**(A)** 11 K emission spectra of CeNR (red), CeNR-PhEt-BuOOH (green) and CeNR-H_2_O_2_ (magenta) excited at 316 nm: the dotted lines represent spectral deconvolution using two Gaussian functions attributed to the *5d → ^2^ F_5/2,7/2_* transitions. The inset shows the emission spectra of CeNR (black) and CeNR600 (cyan) excited at 332 nm. **(B)** Emission intensity as a function of temperature of CeNR (red squares) and the Mott-Seitz fitting (solid line) using Equation. (8).

The emission spectra at -262 °C of CeNR, CeNR-H_2_O_2_ and CeNR-PhEt-BuOOH (excitation at 332 nm) display two overlapping broad bands at *ca.* 375 and 410 nm, exemplified for CeNR-H_2_O_2_ by spectral deconvolution (Figure 8A). In contrast to that observed for CeNT-H_2_O_2_, in the case of CeNR and CeNR-PhEt-BuOOH, a series of sharp peaks at *ca.* 370.1, 375.8, 380.6, 386.3 and 390.5 nm were discerned superimposed to the broad band envelope. Whereas the two broad bands are unequivocally (because their energy separation is 2270 cm^-1^) attributed to *Ce^3+^ 5d → ^2^ F_5/2,7/2_* transitions [33,34], the nature of the sharp peaks is still open to discussion: some authors suggest that the peaks are attributed to the hopping from localized Ce^3+^ states or different defect levels to the *O 2p* band [35].

The emission spectra of CeNR and CeNR600 excited at 332 nm are displayed in Figure 8 (inset) showing the same sharp peaks (360–400 nm) associated with the hopping from localized Ce^3+^ states or different defect levels to the *O 2p* band superimposed to the *5d → ^2^ F_5/2,7/2_* broad transitions. Although the emission spectra of all the different materials clearly show the presence of Ce^3+^ ions, the differences between the relative intensity of the sharp peaks and the *5d → ^2^ F_5/2,7/2_* and *4f → O 2p* broad transitions when comparing CeNR600 to the non-calcined samples suggest that the calcination process modifies the distribution of Ce^3+^ ions present in bulk regions and/or in surface defects [19]. These results together with the catalytic ones, suggest that the presence of Ce^3+^/Ce^4+^ in the material is not sufficient for the existence of catalytic activity and most likely catalytic activation is associated to a certain population of defect sites.

Emission spectra were acquired as a function of temperature, wavelength excitation of 282 nm, and the evolution of the integrated emission is depicted in Figure 8B. At room temperature, CeNR material does not emit. Several reasons have been put forward to explain the quenching of luminescence including the thermally-excited interconfigurational system crossing, energy transfer from *4f* level to nearby centres, electron transfer or photoionization [36]. Between -262 and -173 °C the CeNR emission intensity remains constant and then it decreases markedly, almost disappearing at *ca.* -53 °C. This could be the result of defects (including oxygen vacancies site in the crystal) with electronic energy levels between the O*2p* and 4*f* bands. The number of these defects is temperature dependent [37,38,39]. This behaviour can be described by a thermally-activated non-radioactive mechanism modelled by the Mott Seitz Equation [40,41]

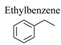
(8)
where *a* is a constant and *ΔE* is the activation energy that corresponds to the energy gap between the *4f* level and that of the de-excitation states. A fit of the experimental values by Equation (8) (Figure 8B) gives an activation energy of *ca.* 651 cm^-1^ (81 meV). This value is consistent with a previous report where the defect levels are located in the range of 1 eV below the Ce *4f* band [38,39].

## 3. Experimental Section

### 3.1. Samples

Cerium oxide nanorods (CeNR) were obtained by hydrothermal synthesis using a published procedure [8]: some nanotubes were formed (ascertained by TEM analysis as discussed above), albeit apparently they represent a minor fraction of this sample. In a typical reaction, 0.6 g of Ce_2_(SO_4_)_3_.9H_2_O (Aldrich) was dispersed in 35 cm^3^ of NaOH (12 M). This mixture was stirred during 1 h, then transferred to a Teflon^®^ autoclave (inner volume 42 cm^3^) and placed in a pre-heated furnace at 135 °C for 15 h. The yellowish Ce(OH)_3_ precipitates were then filtered, washed and dried at 60 °C under nitrogen atmosphere. The materials were left under air for a week giving CeNR.

The CeNR material was partially oxidised in air, at room temperature, for 24 h and then dispersed in distilled water and treated ultrasonically for 2 h with 70% aqueous tert-butyl hydroperoxide (t-BuOOH) or 15% aqueous hydrogen peroxide to give CeNR-BuOOH and CeNR-H_2_O_2_, respectively.

### 3.2. Structure and Texture Characterisation

TEM measurements were done on H-9NA microscope over the ceria samples on amorphous carbon-Fomvar^®^ grid support. These samples were previously dispersed in distilled water (0.5 mg/mL) with ultrasonic stirring during 10 min; the TEM grid was deep coated in this suspension and dried in air.

The XRD patterns were recorded using a Philips X’Pert MPD powder X-ray diffractometer. The samples were exposed to the CuK_α_ radiation (1.54 Å) in a 2θ range between 1 and 80º with a step of 0.04 and acquisition time of 40 s per step.

The nitrogen adsorption-desorption isotherms were measured at -196 °C, using a Micromeritics Gemini^®^ equipment. Prior to analysis the powdered samples were pre-treated at 150 °C. The BET specific surface areas were calculated for p/p_0_ in the range 0.015–0.15. The pore size distribution (PSD) curves were calculated from the desorption branches of the isotherms using the Barrett-Joiner- Halenda method.

### 3.3. Catalysis

The liquid-phase oxidation of ethylbenzene was carried out under air and autogeneous pressure, in the temperature range 55–105 °C, using a micro reactor equipped with a valve for sampling, a magnetic stirrer and immersed in a thermostated oil bath. The micro vessel was loaded with 30 mg of catalyst, 1.5 mmol of substrate, 3 mmol of oxidant (70% aq. tert-butyl hydroperoxide or 30% aq. H_2_O_2_) and 1.5 cm^3^ of acetonitrile (is of modest toxicity and seems fairly stable under the applied reaction conditions) as solvent. The reaction mixture was cooled in an ice bath during 30 seconds prior to opening the valve for sampling, which was performed under stirring. Cyclohexanol and cyclohexene were also tested as substrates. Samples were analysed using a Varian 3800 gas chromatograph equipped with a DB-5 30 m ° 0.25 mm capillary column and a flame ionisation detector, using decane as internal standard added to the withdrawn samples. The reaction products were identified by GC-MS (HP 5890 Series II GC; HP 5970 Series Mass Selective Detector) using He as carrier gas.

### 3.4. UV/Vis Spectroscopy

UV/Vis absorbance spectra were acquired from CeNR, CeNR-BuOOH, CeNR-H_2_O_2_ and CeNR-PhEt-BuOOH powders and from CeNR dispersion on a Jasco V 560 UV/Vis spectrometer. The absorbance spectrum from CeNR dispersion was recorded in a quartz cell (1 cm path length), and hexane or ethanol were used as blank. The optical absorption coefficient α was calculated according to α = (Aρ)/lc 13], where A is the absorbance of the sample, ρ the density of CeO_2_ (7.28 g cm^-3^), l the path length (1 cm), and c the concentration of the ceria suspension (0.00026 g cm^-3^).

### 3.5. Photoluminescence Spectroscopy

Emission and excitation spectra were recorded between -262 °C and room temperature on a Fluorolog^®^-3 Model FL3–2T with double excitation spectrometer (Triax 320), fitted with a 1200 grooves mm^-1^ grating blazed at 330 nm, and a single emission spectrometer (Triax 320), fitted with a 1200 grooves/mm grating blazed at 500 nm, coupled to R928 photomultiplier. Excitation spectra were corrected from 240 to 580 nm for the spectral distribution of the lamp intensity using a photodiode reference detector. Emission spectra were also corrected for the spectral response of the monochromators and the detector using typical correction spectra provided by the manufacturer.

## 4. Conclusions

The CeO_2_ nanorods (CeNR) promote the oxidation of ethylbenzene (PhEt) and cyclohexene with *t*-BuOOH, at temperatures as low as 55 °C. The reaction of PhEt gives 1-phenyl-ethyl-*tert*-butyl-peroxide (39% yield after 48 h, 55 °C), which may be converted into acetophenone (70% selectivity at 89% conversion, 105 °C). The reaction of cyclohexene with *t*-BuOOH, at 55 °C, gives mainly 1-(*tert*-butyl-peroxy)-2-cyclohexene (86% selectivity at 22% conversion). For both substrates the saturated C-H bonds are preferentially activated over the unsaturated ones.

The liquid-phase oxidation catalysis with CeNR/*t*-BuOOH may be associated with the Ce^3+^/Ce^4+^ inter-conversion in the one-electron redox processes mediating the formation of *tert*-butyl-(per)oxy radicals. The *tert*-butyl-peroxy products of the reactions of cyclohexene and ethylbenzene are most likely formed *via* the homolytic addition of *tert*-butyl-peroxy radicals (these results contribute to the list of the main applications of *t*-BuOOH as a source for *tert*-butyl derivatives, including alkyl peroxides). CeNR is fairly stable towards leaching of cerium and the catalytic results obtained for the used CeNR are similar to those observed in the first batch run.

Pre-treatment of CeNR with H_2_O_2_ or *t*-BuOOH prior to reaction of PhEt with *t*-BuOOH leads to faster reaction than CeNR. While ATR-FTIR does not show major differences in the bulk features of CeNR (before and after exposure to the oxidants), UV-vis and photoluminescence spectroscopies shows noticeable differences in the case of CeNR-H_2_O_2_, which are probably associated to the defect sites present in the materials (supported by the powder XRD data and TEM images): UV-vis and photoluminescence spectroscopy confirm the presence of both Ce^3+^ and Ce^4+^ in all investigated materials. Thermal treatment at 600 °C of CeNR has a detrimental effect on catalytic activity. While the powder XRD and TEM data are similar for CeNR and CeNR600, the emission spectra of these materials shows changes in the distribution of Ce^3+^/Ce^4+^ ions probably in defect sites. Although exposure to H_2_O_2_ seems to give a more defective material (CeNR is very effective in the disproportionation of H_2_O_2_) than that with *t*-BuOOH, the PhEt reaction is faster for CeNR treated with *t*-BuOOH. The enhanced catalytic activity associated with the generation of defect sites may be levelled off by the concomitant morphological changes (extensive fragmentation) leading to defect sites with different structures and electronic properties (and expectedly different intrinsic catalytic activity). A detailed study using XPS spectroscopy could give further insights on structure-activity relationships.
